# Iron Deficiency Impairs Intra-Hepatic Lymphocyte Mediated Immune Response

**DOI:** 10.1371/journal.pone.0136106

**Published:** 2015-08-19

**Authors:** Eliano Bonaccorsi-Riani, Richard Danger, Juan José Lozano, Marta Martinez-Picola, Elisavet Kodela, Roser Mas-Malavila, Miquel Bruguera, Helen L. Collins, Robert C. Hider, Marc Martinez-Llordella, Alberto Sanchez-Fueyo

**Affiliations:** 1 Department of Liver Studies, Division of Transplantation Immunology & Mucosal Biology, Medical Research Council (MRC) Centre for Transplantation, Faculty of Life Sciences & Medicine, King's College London University, King's College Hospital, Denmark Hill, London, United Kingdom; 2 Liver Unit and Bioinformatic platform, CIBEREHD, Hospital Clinic Barcelona, Villaroel 170, Barcelona, Spain; 3 Department of Immunobiology, Division of Immunology, Infection & Inflammatory Disease, Faculty of Life Sciences & Medicine, King's College London, Franklin-Wilkins Building, Stamford Street, London, United Kingdom; 4 Institute of Pharmaceutical Science, Faculty of Life Sciences & Medicine, King's College London, Franklin-Wilkins Building, Stamford Street, London, United Kingdom; French National Centre for Scientific Research, FRANCE

## Abstract

Hepatic expression of iron homeostasis genes and serum iron parameters predict the success of immunosuppression withdrawal following clinical liver transplantation, a phenomenon known as spontaneous operational tolerance. In experimental animal models, spontaneous liver allograft tolerance is established through a process that requires intra-hepatic lymphocyte activation and deletion. Our aim was to determine if changes in systemic iron status regulate intra-hepatic lymphocyte responses. We used a murine model of lymphocyte-mediated acute liver inflammation induced by Concanavalin A (ConA) injection employing mice fed with an iron-deficient (IrDef) or an iron-balanced diet (IrRepl). While the mild iron deficiency induced by the IrDef diet did not significantly modify the steady state immune cell repertoire and systemic cytokine levels, it significantly dampened inflammatory liver damage after ConA challenge. These findings were associated with a marked decrease in T cell and NKT cell activation following ConA injection in IrDef mice. The decreased liver injury observed in IrDef mice was independent from changes in the gut microflora, and was replicated employing an iron specific chelator that did not modify intra-hepatic hepcidin secretion. Furthermore, low-dose iron chelation markedly impaired the activation of isolated T cells *in vitro*. All together, these results suggest that small changes in iron homeostasis can have a major effect in the regulation of intra-hepatic lymphocyte mediated responses.

## Introduction

Iron homeostasis and the immune system are closely interconnected. Inflammatory cytokines induce a rapid increase of hepcidin [1], the central mediator of iron homeostasis, which reduces iron export from enterocytes, hepatocytes and, most importantly, macrophages [2]. This results in iron accumulation within macrophages and decreased circulating iron levels, which appears to be an effective defence strategy against extracellular microorganisms that need access to iron to exert pathogenic effects [2]. On the other hand, iron loaded macrophages exhibit reduced effector functions, which can compromise their capacity to clear intra-cellular infections [3, 4]. Iron also contributes to the formation of potent reactive radicals, which promote oxidative stress and induce inflammation and tissue injury. Thus, iron restriction has been shown to ameliorate oxidative stress and inflammatory organ damage in models of murine acute hepatitis [5, 6], experimental autoimmune encephalomyelitis (EAE) [7], renal interstitial fibrosis [8] and type 2 diabetes in rats [9]. In addition to its effects on innate immune responses and inflammation, iron is also required for the function and differentiation of adaptive immune cells such as T lymphocytes. Thus, iron deficiency impairs T cell proliferation *in vitro* [10]. The impact of iron homeostasis manipulations on lymphocyte function *in vivo*, however, has not been investigated in detail.

A role for iron homeostasis in the regulation of intra-hepatic lymphocyte responses and in the development of transplantation tolerance has been recently suggested on the basis of the results of a clinical trial in which immunosuppressive drugs were intentionally withdrawn from a cohort of 98 long-term surviving liver transplant recipients [11]. As compared to tolerant liver recipients who successfully discontinued immunosuppression and age-matched healthy individuals, non-tolerant patients who rejected during the weaning process exhibited lower serum hepcidin and ferritin levels, as well as lower intra-hepatic iron content and hepcidin gene expression. An intra-hepatic iron-related gene expression signature was indeed the most accurate marker to predict the outcome of the drug withdrawal protocol. The phenomenon of spontaneous liver allograft tolerance has been extensively studied in experimental animal models, particularly in rodents, in which an essential step in the induction of tolerance is the activation and subsequent deletion of liver infiltrating lymphocytes [12, 13]. On account of these data, we hypothesized that small changes in iron homeostasis could influence intra-hepatic immune responses by interfering with intra-hepatic lymphocyte activation. To test this hypothesis, in the current study we induced mild iron deficiency in mice by administering either an iron-free diet or low-dose iron chelators, and assessed how iron deficiency influenced intra-hepatic lymphocyte activation and its downstream effects. Reduction of iron availability significantly interfered with lymphocyte activation, proliferation and cytokine production and resulted in dampened immune-mediated hepatitis. These findings highlight a previously not well-recognized phenomenon through which small changes in iron homeostasis can influence outcomes in inflammatory liver disorders.

## Material and Methods

### Mice

Male C57Bl/6 mice (purchased from Charles River) were bred under specific pathogen-free conditions. All procedures were conducted in accordance with national and institutional guidelines for animal care and use and have been approved by the Denmark Hill Animal Welfare and Ethical Review Body (AWERB–KCL) and the Home Office. Anesthesia was performed by isoflurane inhalation whereas euthanasia was performed in CO_2_ chamber.

Four-week old mice were fed with either iron deficient (<6mg/kg iron) or iron replete (iron deficient diet supplemented with 200 mg/kg iron carbonyl) diets (SAFE, France) for a total of 3 weeks prior to the performance of experiments. In some experiments, mice also received an antibiotic cocktail consisting of ampicillin 1 g/L, vancomycin 0.5 g/L, (Laboratoiros Normon S.A., Spain), metronidazole 1 g/L (BBraun Medical S.A., Spain) and neomycin 1 g/L (Laboratorios Salvat, Spain), which was added to drinking water and changed every 3 days for the entire duration of the 3-week period as described [14].

### Concanavalin A (ConA) induced hepatitis

In order to investigate a potential iron-related functional effect in immune system, we used an immune-modulated model of acute hepatitis using a sublethal injection of Concanavalin A (ConA) [15]. Immune-mediated acute hepatitis was induced by intravenous injection of 15mg/kg of type IV ConA (Sigma-Aldrich, St. Louis, MO). Following ConA injection mice were anesthetized and sacrificed at various time points to collect blood, spleen and liver tissue. Additional experiments were performed with a single previous IP injection of 100μg of mouse hepcidin (PLP-3773, Peptides International, KY, USA) or sterile PBS two hours before the ConA challenge.

### Iron chelators

For some *in vivo* experiments, a 3 day-course of a hydroxypyridinone iron based chelator (HPO CP28; 20 nmoles, i.p.) [16, 17] was performed before ConA challenge. *In vitro*, cells were incubated with low doses of either HPO CP182 (5μM) or desferoxamine (DFO, 10μM, Sigma-Aldrich). At these doses iron chelators had no discernible effects on lymphocyte cell death (S1 Fig).

### Serum biochemistry and cytokine analyses

Serum was obtained after 10 minutes centrifugation at 8,000g and stored at -80C. The alanine aminotransferase (ALT) plasma activities, which reflect the liver injury intensity, and the serum iron level were quantified by automated measurements using the ADVIA 2400 System kits (Siemens). Hematologic parameters were assessed by automated measurements using the ADVIA 2120 System kits (Siemens). Serum cytokine levels were measured with multiplex fluorescent bead-based Luminex technology according to manufacturer’s recommendation.

### Liver histology

Formalin-fixed and paraffin embedded liver tissue sections were stained with haematoxylin and eosin stained and the presence of hepatocyte necrosis, cellular infiltration and hepatocyte cohesion were assessed by a senior liver histopathologist who was blinded to the identity of the experimental groups (MB).

### Isolation of non-parenchymal liver cells

After harvesting, the liver was perfused with sterile PBS through the portal vein, and digested at 37°C in the presence of collagenase II 5%,(PAA Laboratories) and 100μg/mL DNase I (Roche) for 30 minutes, as described [18]. The liver tissue was then passed through a 70μm nylon mesh. Hepatocytes were then removed following low-speed centrifugation (60 xg, 2minutes) and liver mononuclear cells were isolated using Ficoll separation (800 xg, 20 minutes).

### Isolation of splenic and lymph node leukocytes

Spleens were passed through a 70um nylon mesh, and blood cells were removed using ammonium chloride potassium lysing buffer (Life Technologies) for 5 minutes.

### *In vitro* naïve CD4^+^ T cell stimulation

CD4^+^ naïve T cells were isolated from spleen and lymph nodes using EasySep Mouse Naïve CD4^+^ T Cell Isolation Kit (Stem Cell). Enriched cells were labelled with 2.5 μM CFSE (Biolegend) during 10 minutes. A total of 250.10^3^ cells were incubated during 5 days in 96 well-plate coated with anti-CD3 anti-CD28 antibodies (2ug each, clone 145-2C11 and 37.51, respectively) in RPMI-1640 (Life technologies) supplemented with 5% Foetal Calf Serum (Sigma-Aldrich), 1% penicillin-streptomycin and 2 mM L-glutamine and 50μM β-mercaptoethanol (Life technologies).

### Flow cytometry

Up to 1.10^6^ cells were labelled in staining solution (PBS, Foetal Calf Serum 2%, 2mM EDTA). Antibodies used were: APC/Cy7 anti-CD3e, PE/Cy7 anti-CD4, APC anti-CD25, PE anti-CD44, FITC anti-CD62L (all from Biolegend). Dead cells were stained either with 7-AAD (Biolegend) or Livedead cell viability assay (Life Technologies). Cells were fixed using fixation solution 20 minutes at room temperature (Biolegend). For regulatory T cells labelling, FOXP3 Perm/Fix solution was use with PE anti-FOXP3 antibody (Biolegend) according to manufacturer’s instructions. Intracellular IFNγ staining was performed using permeabilisation buffer following manufacturer’s instructions and FITC anti-IFNγ antibody and APC anti-IL4 antibody (Biolegend). Data were acquired on a BD FACSCanto II flow cytometer (BD) and analyzed employing FlowJo software (Tree Star).

### RNA extraction and gene expression experiments

Liver or spleen tissue samples were cryopreserved at -80C in RNAlater reagent (Ambion). For RNA extraction, samples were first homogeneized using a RNAse-free pestle in Trizol reagent (Invitrogen), and total RNA was extracted according to TRIzol manufacturer’s protocol. DNA was removed from total RNA preparations using Turbo DNA-free DNAse treatment (Ambion). Quality and quantity were assessed with the Agilent 2100 Bioanalyzer (Agilent Technologies) and Nanodrop ND-1000, respectively. Total RNA was then reverse transcribed into cDNA using the High-Capacity cDNA Reverse Transcription Kit (Applied Biosystems). A pre-amplification of cDNA was performed using pooled TaqMan Assays (final concentration of 0.2X each) and the TaqMan PreAmp Master Mix using 10 cycles of amplification. qPCR was performed using the 48.48 Dynamic Array (Fluidigm Corporation, CA, USA) following manufacturer's protocol using a BioMark Instrument (Fluidigm Corporation). To quantify transcript levels, target gene Ct values were normalized using Ct values of HPRT1 as a reference gene to generate -ΔCt values. Real-time PCR gene expression experiments were performed at 12 hours post injection. This time point was selected to coincide with the peak of transaminases and after determining that it required more than 3 hours to detect significant gene expression differences (in a pilot experiment, Illumina microarrays performed at 3 hours showed minimal differential gene expression (data not shown)).

### Statistical analyses

Statistical analyses were performed with GraphPrism software. Student T-test was used for comparison between two groups and ANOVA analysis with Tukey’s post-hoc correction for pairwise comparisons was used to compare more than 2 groups. Fisher test was used for categorical variables. P-values <0.05 (two-tailed) were considered statistically significant in all analyses.

## Results

### Induction of mild iron deficiency by dietary manipulation

**Fig 1:**
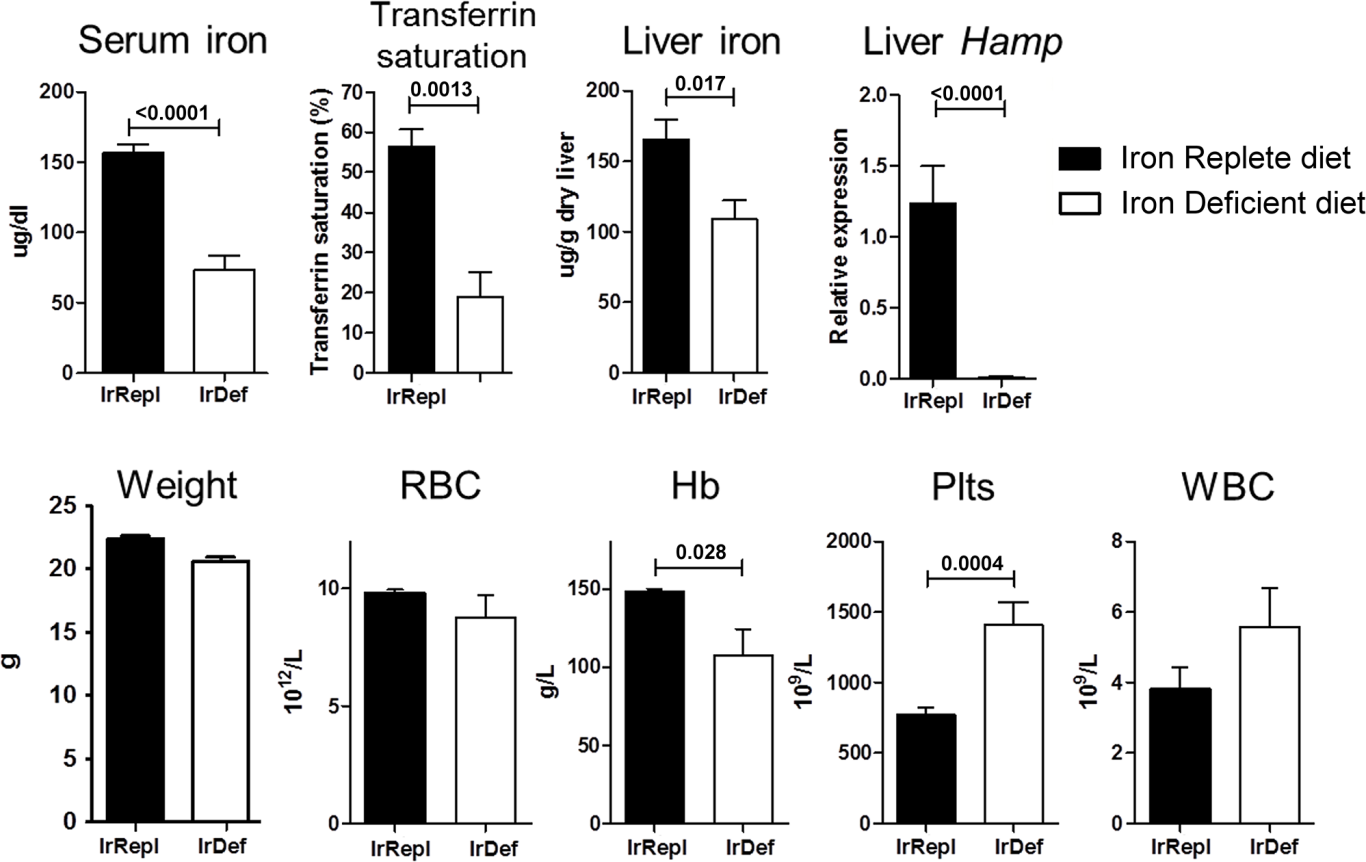
Iron and haematological parameters in mice fed with an iron-deficient diet. Mice were fed for 3 weeks with an iron deficient (IrDef) or iron replete (IrRepl) diet. Bar plots display mean and SEM. Abbreviations: RBC (red blood cells), Hb (Hemoglobin), Plts (platelets), WBC (white blood cells).

As compared to mice fed with an iron replete diet (IrRepl), mice fed with an iron deficient diet (IrDef) displayed decreased serum iron (mean 73.22 ±56.49 *versus* 156.38 ±36.99 μg/dL; p <0.0001) and serum transferrin saturation (18.80 ±6.32 vs 56.20 ±4.53%; p = 0.0013) (Fig 1). In addition, liver tissue analysis revealed reduced liver iron stores and hepcidin (*Hamp*) gene expression (p = 0.017 and p<0.0001, respectively) (Fig 1). None of these iron parameters were found different between IrRepl diet and regular diet. As previously reported [19, 20], IrDef mice also developed mild anemia (hemoglogin 107.6 ±46.10 versus 147.9 ±6.50 g/L; p = 0.028), and increased platelet count (1405 ±469.2 versus 769.4 ±174.4 x10^9^/L; p = 0.0004) (Fig 1). IrDef mice remained otherwise healthy and gained weight at the same rate as IrRepl mice.

### Mild iron deficiency does not modify the steady state immune cell repertoire and systemic cytokine levels

**Fig 2:**
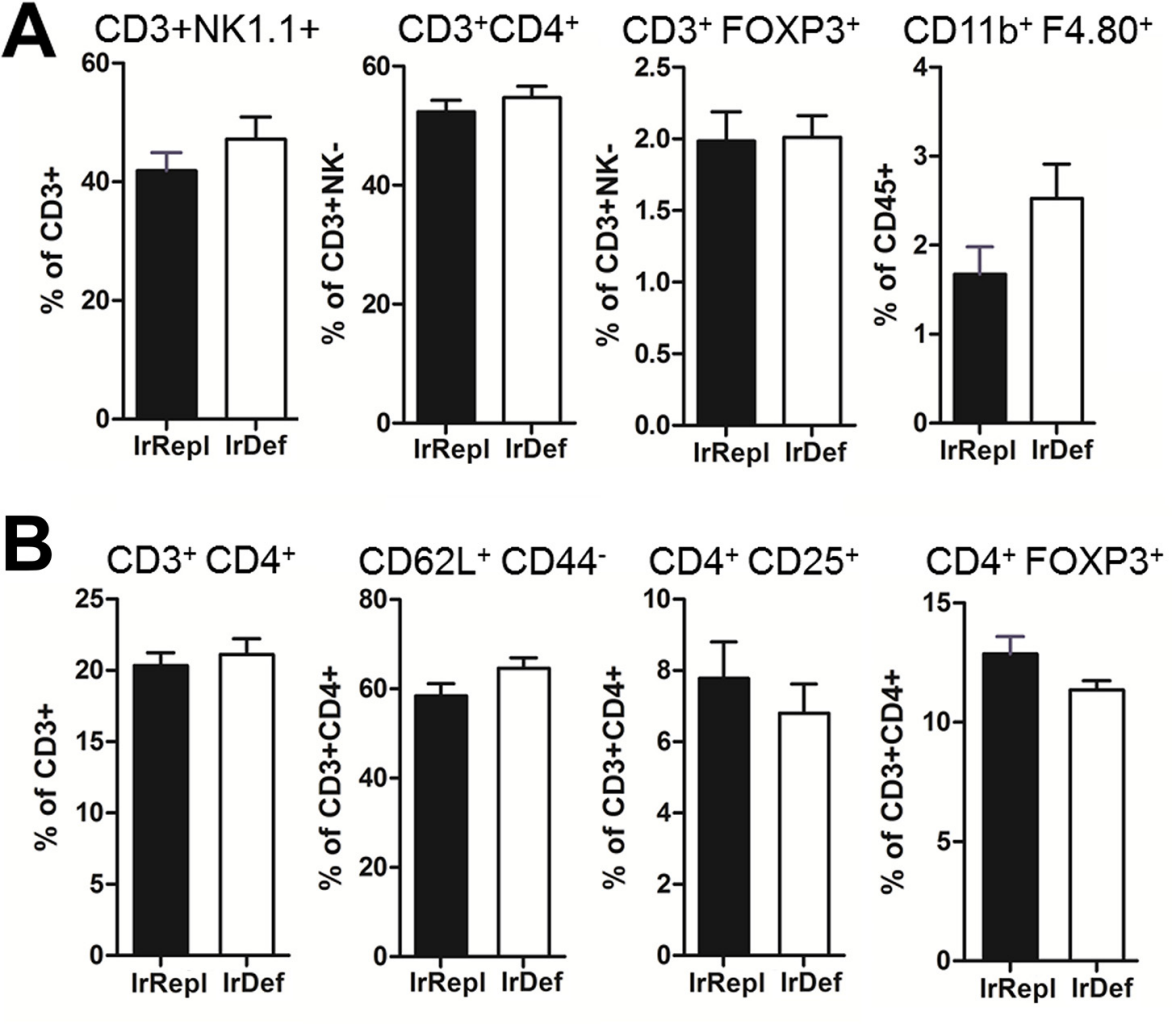
Influence of iron levels on the immunophenotype of intra-hepatic (A) and spleen (B) leukocytes. Mice were fed for 3 weeks with an iron deficient (IrDef) or iron replete (IrRepl) diet. Bar plots show the frequency (mean and SEM) of specific leukocyte subsets in the liver (A) and spleen (B), as assessed by flow cytometry after excluding dead cells.

To assess the impact of mild iron deficiency induced by an iron deficient diet on immune parameters, we first compared liver and spleen immune cell subsets in IrDef and IrRepl mice. The 2 groups of mice displayed similar frequencies of intra-hepatic NK1.1^+^CD3^+^ NKT, CD4^+^ and CD8^+^ T cells, FOXP3^+^ T cells, cells and resident CD45^+^CD11b^+^F4.80^+^ Kupffer cells (Fig 2A). Similarly, no differences between IrDef and IrRepl splenic T cell subsets were noted, suggesting that in the steady state IrDef does not induce major immunological effects (Fig 2B). We next explored the effects of the iron deficient diet on pro-inflammatory and immunoregulatory genes by measuring the expression of interleukins *Il2*, *Il4*, *Il6*, *Il10*, *Il15*, tumor necrosis factor α (*Tnf*), transforming growth factor β (*Tgfb1*), interferon γ (*Ifng*), *Cd86*, forkhead box P3 (*Foxp3*), and *Cd274* in whole livers, spleens and mesenteric lymph nodes from IrDef and IrRepl mice. No significant changes were observed except for small decreases of *TNFα* (fold change (FC) = -0.36, p = 0.032) and *IFNγ* (FC = -0.72, p = 0.044) transcript levels in the liver and spleen of IrDef mice, respectively (Table 1). Serum cytokine levels were also measured using Luminex technology, and again no significant changes between IrDef and IrRepl mice were observed (Table 2).

**Table 1:** Gene expression in liver, spleen and mesenteric lymph nodes (mesLN) of immune-related molecules from IrDef and IrRepl mice. FC is exhibited as Log_2_ of IrDef/IrRepl values. Genes with significant differential expression are highlighted in bold.

		Liver		Spleen		mesLN	
Symbol	Name	FC	p.value	FC	p.value	FC	p.value
*Il2*	Interleukin 2	0.75	0.090	-0.49	0.20	-0.26	0.28
*Il4*	Interleukin 4	0.32	0.14	-0.038	0.90	0.24	0.50
*Il6*	Interleukin 6	-0.20	0.39	-0.094	0.70	-0.48	0.15
*Il10*	Interleukin 10	-0.15	0.48	-0.11	0.74	0.060	0.83
*Il15*	Interleukin 15	0.098	0.68	-0.38	0.18	0.36	0.13
*Ifng*	Interferon gamma	0.31	0.26	**-0.72**	**0.044**	0.40	0.54
*Tnf*	Tumor necrosis factor	**-0.36**	**0.032**	-0.29	0.38	0.024	0.94
*Tgfb1*	Transforming growth factor, beta 1	0.17	0.14	-0.26	0.14	-0.12	0.53
*Foxp3*	Forkhead box P3	-0.046	0.88	-0.32	0.34	0.052	0.85
*Cd86*	CD86 antigen	0.003	0.97	-0.41	0.083	-0.24	0.26
*Cd274*	CD274 antigen	0.10	0.38	-0.40	0.10	0.020	0.95

**Table 2:** Serum level of cytokines measured by Luminex technology from IrDef and IrRepl mice. FC is exhibited as Log_2_ of IrDef/IrRepl values.

Symbol	Name	FC	p.value
IFNy	Interferon gamma	0.85	0.59
IL-1α	Interleukin 1alpha	-0.73	0.57
IL-2	Interleukin 2	-3.35	0.20
IL-4	Interleukin 4	0.90	0.48
IL-6	Interleukin 6	-0.48	0.68
IL-10	Interleukin 10	-1.11	0.26
IL-15	Interleukin 15	1.47	0.10
IL-17	Interleukin 17	-1.69	0.15
CXCL10	chemokine (C-X-C motif) ligand 10 (IP-10)	0.008	0.96
CXCL9	chemokine (C-X-C motif) ligand 9 (MIG)	0.41	0.61
TNF α	Tumor necrosis factor alpha	-0.27	0.51

### Mild iron deficiency significantly dampens inflammatory liver damage following ConA injection

**Fig 3:**
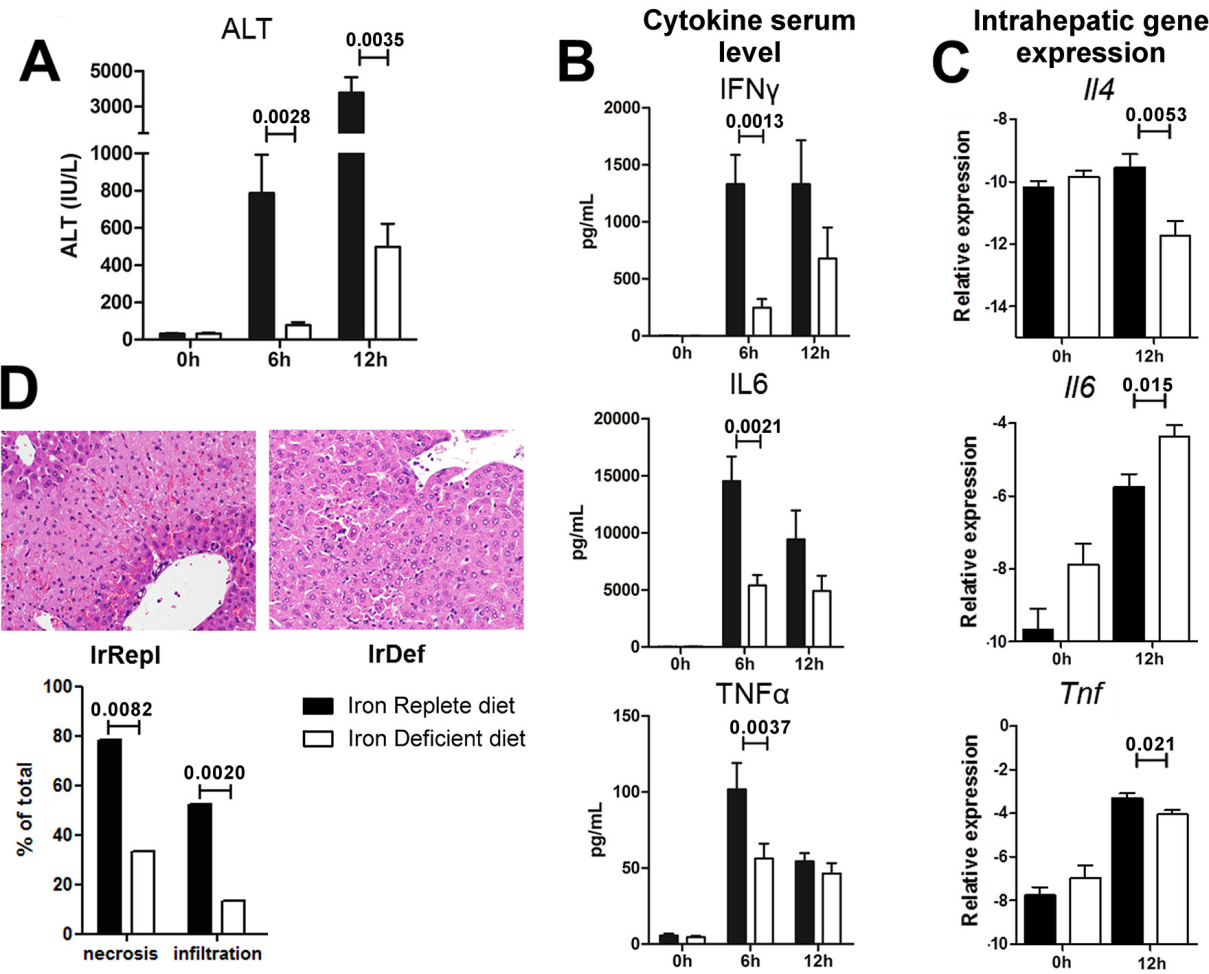
Iron deficiency results in attenuated immune-mediated hepatitis following ConA administration. (A) Serum levels of alanine aminotransferase (ALT; IU/mL) before, 6 hours and 12 hours after administration of ConA in mice fed for 3 weeks with an iron deficient (IrDef) or iron replete (IrRepl) diet. (B) Cytokine levels in serum samples collected before, 6 hours and 12 hours after the administration of ConA. (C) Transcript levels of *Il4*, *Il6 and TNFα* in liver tissue samples collected before and 12 hours after the administration of ConA. (D) Representative liver histology (200x) at 12 hours after the administration of ConA (upper panel) and histologic evaluation of necrosis and infiltration (lower panel).

To determine the functional effect of mild iron deficiency on intra-hepatic lymphocyte responses we employed the ConA-induced immune-mediated hepatitis model. Following ConA injection, the rise of alanine aminotransferases (ALT) was significantly lower in IrDef mice than in IrRepl mice (p = 0.0028 and 0.0035 at 6 and 12 hours after ConA administration, respectively) (Fig 3A). This was associated with reduced serum levels of the pro-inflammatory cytokines IFNγ, IL6 and TNFα, which reached statistical significance at 6 hours after ConA injection (p = 0.0037, 0.0013 and 0.0021, respectively; Fig 3B). Similar changes were observed in the intra-hepatic expression of *Il4 and Tnfa*, whose transcript levels significantly decreased in IrDef mice 12 hours after ConA injection (p = 0.0053 and p = 0.021, respectively; Fig 3C). These data were consistent with the results of the liver histopathology analysis, which revealed decreased hepatocyte necrosis and inflammatory infiltration in IrDef as compared to IrRepl mice (p = 0.0082 and 0.0020, respectively) (Fig 3D).

### Reduced iron availability impairs lymphocyte proliferation and activation

**Fig 4:**
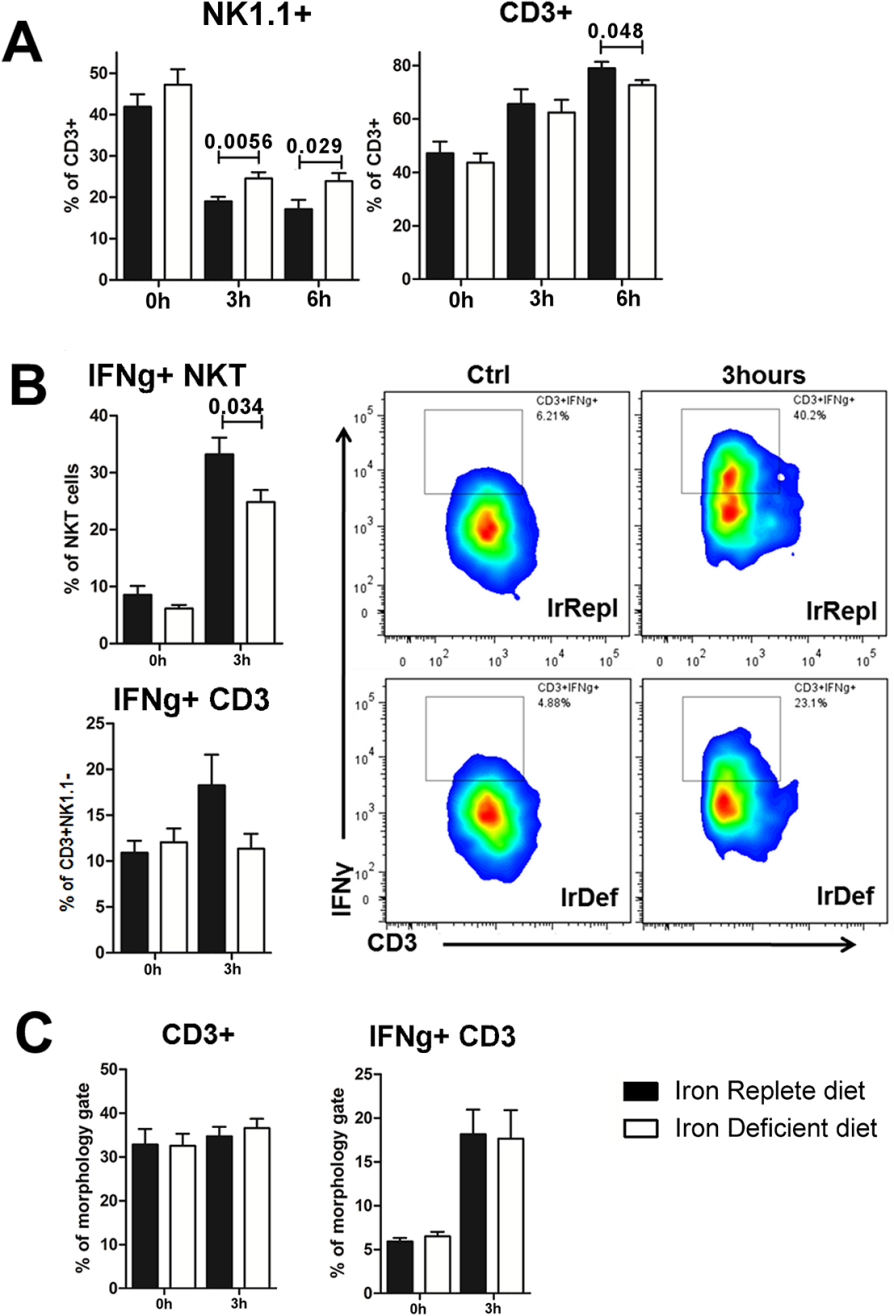
ConA-induced T and NKT lymphocyte activation is reduced in iron deficiency. (A) Intra-hepatic frequency of CD3^+^ expressing NK1.1 and CD3^+^NK1.1^-^ at baseline, 3 hours and 6 hours after ConA injection. (B) Intra-hepatic frequency of IFNγ production by NKT cells and T cells, defined as CD3^+^NK1.1^+^ and CD3^+^NK1.1^-^, respectively, (left panel) and representative staining of IFNγ production by NKT cells (right panel) (C) Frequency of splenic CD3^+^ NK1.1^-^ T cells and the percentage of these ones producing IFNγ.

ConA administration triggers an inflammatory cascade that requires activation of T and NKT lymphocytes and eventually results in liver tissue damage [15, 21]. Following ConA activation, lymphocytes produce IFNγ, which is directly involved in the pathogenesis of ConA-induced hepatitis [21–23]. Furthermore, NKT cells down-regulate NK1.1 and CD3 surface markers, and the expression of these markers is inversely correlated to the degree of NKT activation [24]. To explore if the effects of iron deficiency are mediated by defective lymphocyte activation, we isolated spleen and intra-hepatic lymphocytes at different time points following ConA administration and assessed their phenotypic and functional properties. In IrDef mice, ConA induced significantly less accumulation of liver-infiltrating CD3^+^ lymphocytes than in IrRepl mice (p = 0.048 at 6 hours; Fig 4A). This was associated with a less striking decrease in the expression of NK1.1 among CD3 cells (a marker of NKT cell activation) both at 3 and 6h following ConA injection (p = 0.0056 and 0.029, respectively; Fig 4A). In addition, as compared to IrRepl, 3h after ConA administration intra-hepatic NKT and T lymphocytes from IrDef displayed lower intra-cellular IFNγ levels (p = 0.034 and 0.081, respectively; Fig 4B). Interestingly, in contrast to the significant differences observed in the liver compartment, the frequence of IFNγ-producing lymphocytes in the spleen was similar in IrDef and IrRepl mice (Fig 4C).

**Fig 5:**
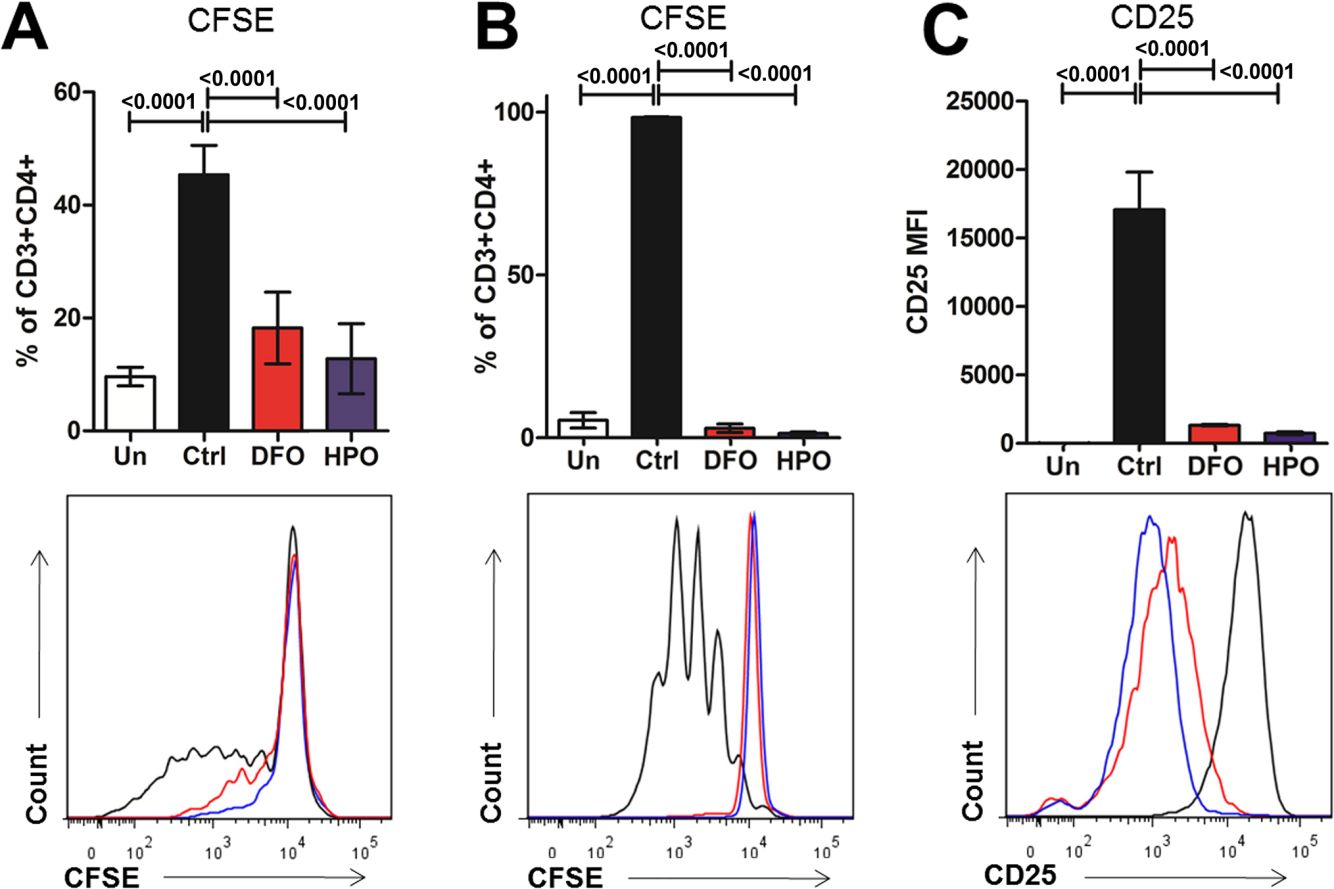
Iron chelation impairs CD4^+^ T cell activation and proliferation *in vitro*. (A) Percentages of CD3^+^CD4^+^ cells from splenocytes undergoing in at least one division are represented with representative fluorescent histogram in the lower part. Splenocytes were incubated 96 hours in presence of ConA (0.1mg/mL) and in presence of low doses of iron chelator (HPO CP182, 5μM or DFO, 10μM). (B) Percentages of CD3^+^CD4^+^ cells from isolated naive CD4^+^ cells undergoing in at least one division and (C) CD25 MFI are represented are displayed with representative fluorescent histogram in the lower part. Isolated CD4^+^ naïve T cells incubated 5 days in presence of anti-CD3/CD28 plate-bound antibody (2μg each). *Un* means unstimulated cells.

To confirm the effects of iron deficiency on T cells, we incubated splenocytes *in vitro* with ConA (0.1mg/mL) in the presence of low doses of a specific hydroxypyridinone iron chelator [16, 17] (HPO CP182; 5μM) or desferoxamine (DFO; 10μM). Iron chelation resulted in impaired CD3^+^CD4^+^ T cell proliferation (p<0.001). Furthermore, iron chelators also hampered the activation of isolated CD4^+^ naïve T cells, which, following stimulation with plate-bound anti-CD3/CD28, showed decreased CD25 expression and cell proliferation (Fig 5).

### The effects of iron deficiency in intra-hepatic inflammatory responses are independent from changes in the gut microbiota

**Fig 6:**
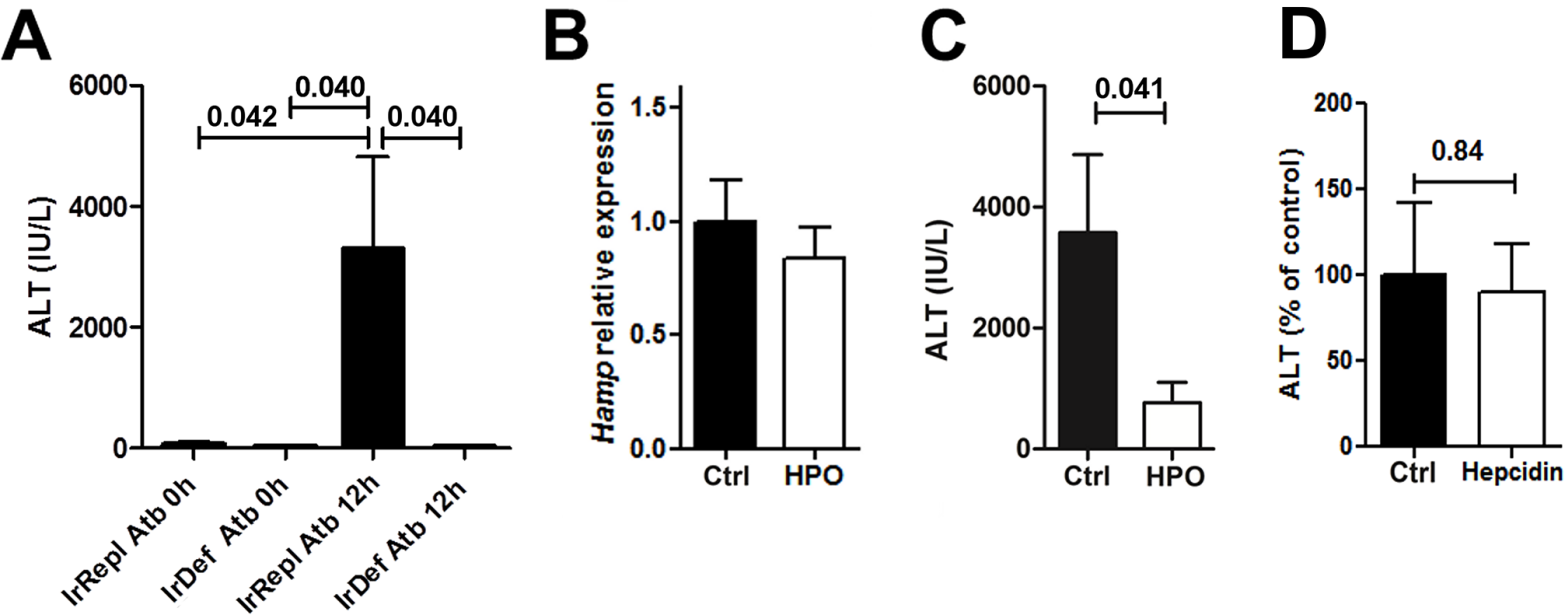
The inhibitory effects of iron deficiency in ConA-induced hepatitis are independent from changes in gut microbiome and hepcidin levels. (A) ALT serum levels (IU/mL) before, and 12 hours after the administration of ConA to mice fed for 3 weeks with an iron deficient (IrDef) or iron replete (IrRepl) diet, in the presence or absence of a 4-antibiotic (Atb) cocktail. (B) Relative expression of *Hamp* in liver tissue samples from mice receiving a 3 day-course of HPO CP28 (20 nmoles, daily) compared to control mice receiving PBS (p = 0.48). (C) ALT serum levels 12 hours after ConA challenge in mice treated with HPO CP28 or with PBS (p = 0.041). (D) ALT serum levels 12 hours after ConA challenge in mice pre-treated two hours before with a single intraperitoneal injection of 100μg of mouse hepcidin or sterile PBS (p = 0.84). Bar plots display mean and SEM.

Given the influence of the gut microbiota on the liver environment [25] and the well-recognized effects of iron levels on bacterial growth and intestinal microbial composition [26], we next investigated if the reduced immune-mediated hepatitis observed in IrDef mice could be mediated by changes in the number of gut bacteria. IrDef and IrRepl mice received a 3-week course of a 4-antibiotic cocktail known to eliminate >90% of the fecal microbial populations, and ConA was injected at the end of the 3-week period. The results were similar to those observed in mice receiving no antibiotics, with ALT levels being significantly reduced in IrDef as compared with IrRepl mice (Fig 6A).

### Intra-hepatic inflammatory responses are influenced by iron but not by baseline hepcidin level

Iron deficiency results in reduced hepcidin expression which in some models can exert direct immune regulatory effects [19]. We sought to determine if the effects of the iron deficient diet on immune-mediated hepatitis could be due to differences in hepcidin levels rather than to changes in iron status. Mice fed with a standard diet were treated with a 3 day-course of the HPO CP28 iron chelator [16, 17] capable of specifically chelating intra-cellular iron without decreasing intra-hepatic hepcidin expression (p = 0.48) (Fig 6B). Similarly to what was observed employing iron deficient diet, mice treated with the iron chelator displayed a significantly reduced ConA-induced hepatitis (p = 0.041) (Fig 6C). In parallel, administration of exogenous hepcidin to IrDef mice 2h prior to ConA injection did not restore the blunted immune-mediated hepatitis induced by ConA (p = 0.84; Fig 6D). Altogether, these results suggest that iron restriction inhibits lymphocyte activation and ConA-induced hepatitis independently from its effects on hepcidin secretion.

## Discussion

In the current study we investigated the impact of small changes in systemic iron status on intra-hepatic lymphocyte-mediated immune responses. Our experiments were prompted by the results of a recently reported multi-centre clinical trial of immunosuppression discontinuation following liver transplantation [11, 27], in which liver recipients who developed rejection exhibited lower iron storage markers (*e*.*g*. serum ferritin and serum and intra-hepatic hepcidin) than recipients who successfully discontinued immunosuppression (operationally tolerant recipients). These differences were statistically significant and very reproducible, but small in magnitude and not clinically apparent. Given that spontaneous liver allograft tolerance is known to require activation of recipient alloreactive T cells prior to lymphocyte deletion [12, 13, 28], we hypothesized that the iron/hepcidin axis could play an unappreciated role in the regulation of intra-hepatic lymphocyte activation and function.

To test this hypothesis we selected a well-established model of immune-mediated hepatitis that is tightly controlled by the interplay between cytokines, lymphocytes and Kupffer cells, and in which intra-hepatic T/NKT cell activation is necessary for inflammatory liver damage to occur [15, 21, 23]. We replicated the mild iron homeostasis changes observed in the cohort of liver transplant recipients by feeding mice with an iron-deficient diet for just 3 weeks or by administering low-dose iron-specific chelators. Short-term reduction in iron intake did not have a significant impact on the hepatic steady-state immune homeostasis. On the other hand, the magnitude of liver damage upon ConA stimulation was significantly reduced in iron deficient mice, as assessed by decreased serum ALT levels, hepatic necrosis and intra-hepatic cellular infiltration. This was associated with a decrease in systemic levels of pro-inflammatory cytokines such as IL6, TNFα and IFNγ, and with reduced intra-hepatic transcript levels of *IL4* and *Tnfα*, all of them known to regulate ConA immune-mediated hepatitis.

A beneficial effect of dietary iron restriction or DFO iron chelation on liver inflammation was previously reported in experimental animal models of thioacetamide-induced toxic hepatitis [5] and Fas-induced fulminant hepatitis [6]. The anti-inflammatory effects of iron restriction were attributed to a reduction in oxidative stress, and a decreased Kupffer and hepatic stellate cell activation. While oxidative stress and macrophage activation are certainly involved in amplifying liver damage following ConA administration, our findings that iron deficiency results in decreased intra-hepatic T/NKT lymphocyte infiltration and reduced lymphocyte activation and effector function indicates that iron deficiency can also influence the outcome of immune-mediated hepatitis by directly inhibiting intra-hepatic lymphocyte function. These results were confirmed in *in vitro* experiments in which isolated T cells were stimulated with either ConA or anti-CD3/anti-CD28. In these experiments, iron chelation resulted in impaired T cell activation and proliferation.

Our observations indicating that changes in iron status can regulate inflammatory organ damage by influencing lymphocyte activation and proliferation are consistent with results obtained in experimental autoimmune encephalomyelitis, a CD4^+^-driven model in which iron deficiency prevents the development of immunopathology [7]. The mechanisms responsible for T lymphocyte inhibition still remain to be fully deciphered. Intracellular iron deprivation can impair the function of various enzymes involved in cell-cycle control, such as the ribonuleotide reductase [29], involved in DNA synthesis during phase S of cell cycle, cyclin A, and Cdc2 [30]. Iron restriction can also inhibit the hydrolysis of phosphatidyl inositol-4,5-biphosphate and the activity of kinase C protein, both of which are key in the cascade of intracellular signalling events elicited by T cell activation [10, 31]. In addition, at least in the central nervous system, DFO inhibits CXCL10 release following Toll-like receptor 3 (TLR3) engagement [32], which can result in decreased tissue infiltration by CXCR3^+^ T cells.

Given that in some experimental models hepcidin has been reported to be capable of directly inhibiting systemic inflammatory responses independently from iron levels [19, 33], we explored the relative contributions of low iron versus low hepcidin levels in the regulation of ConA-induced immune hepatitis. As expected, iron deprivation resulted in decreased hepatocyte hepcidin expression, which persisted even following the induction of immune-mediated liver damage (data not shown). We investigated the role of low iron levels by administering an iron-specific chelator that does not influence hepatocyte hepcidin expression. Iron chelation replicated the results observed by feeding the animals with an iron deficient diet. We next tested the effect of injecting exogenous hepcidin prior to ConA challenge, which did not modify the inhibitory effects of iron deprivation. We concluded that low iron levels are the major determinant of the reduced liver inflammatory damage observed in iron deficient mice following ConA administration. Considering the effector role played by macrophages in ConA-induced hepatitis, our results appear to be in contrast with observations indicating that iron-deprived macrophages exhibit increased release of pro-inflammatory mediators following activation with LPS [19, 33]. While these observations still need to be clarified, as they have not been confirmed by other studies [34], they suggest that iron deficiency elicits complex immunological effects that are dependent on the specific cell being targeted and on the organ involved in the inflammatory response. Hence, we speculate that the need for effective T cell activation in order for ConA to induce immune mediated liver damage injection, in contrast to what happens when systemic inflammation is provoked by LPS administration, is what determines the different outcomes observed in the 2 models in situations of iron deficiency.

Dietary iron manipulations can modify gut microbiota composition, which may influence liver metabolism and immunogenicity [25]. To exclude this possibility we employed an antibiotic cocktail capable of eliminating >90% of the fecal microbial populations [14]. Iron deficiency was still associated with significantly reduced immune mediated liver damage even in the presence of markedly reduced intestinal bacterial populations. It is noteworthy, however, that as compared with control animals receiving no antibiotic treatment, mice treated with antibiotics exhibited lower transaminase levels following ConA. This was particularly striking in IrDef mice, in which hepatitis was almost completely abrogated. These observations are in agreement with a previous report in which gentamycin decreased ConA-induced liver injury by impairing intestinal dendritic cells function and intra-hepatic NKT cells activation [35]. Thus, in the ConA model, iron deficiency and antibiotic treatment are likely to deliver additive effects by powerfully inhibiting T/NKT cell activation.

While serum Il6 level was decreased after ConA injection in IrDef, we observed an increase of *Il6* expression in the liver. Il6 is commonly described as a pro-inflammatory molecules but our clear read-out of transaminases in the ConA model excludes an exacerbation of liver inflammation. Conversely, Il6 and the subsequent STAT3 phosphorylation have been described to be associated with a reduction of liver injury [36, 37]. The increase *Il6* expression is consistent with a hepatoprotective effect in our setting, but whether IrDef directly participate to this increase of *Il6* expression or this expression is hampered by higher liver damages in IrRepl remains to be determined. The inverse correlation of Il6 levels between serum and liver may reflect the different immunological effects exerted by iron deficiency in the liver as compared with other compartments, or may be the result of an opposite effect exerted on different cell types (*e*.*g*. hepatocytes versus macrophages). Indeed, low hepcidin levels induced by IrDef could be responsible of increased IL6 production by intra-hepatic macrophages as previously described [33].

In conclusion, we report here that iron deprivation impairs intra-hepatic lymphocyte activation and proliferation and results in a beneficial effect on immune mediated hepatitis. Given the central role of the liver in the regulation of iron homeostasis, both as an iron reservoir and as the main source of circulating hepcidin, these findings have implications for a variety of inflammatory liver disorders. Further studies will be needed to ascertain whether all lymphocyte subsets are equally sensitive to iron deprivation, and to determine how iron influences lymphocyte function in situations of chronic antigenic stimulation.

## Supporting Information

S1 Fig percentages of dead cells (7AAD^+^CD4^+^) relative to control in presence of various concentrations of DFO and CP182 with splenocytes activated with CD3/CD28 antibodies during 3 days (1A) and in experiments presented in Fig 5A and 5B and 5C with activated total splenocytes or CD4^+^ naïve T cells, respectively. Representative Annexin V stainings in activated CD4^+^ naïve T cells are exhibited in supplementary figure 1D with no significant differences between groups.
(PPTX)
