# Supplementary figures and images for: Iron Deficiency Impairs Intra-Hepatic Lymphocyte Mediated Immune Response
Source: PLoS One. 2015 Aug 19;10(8):e0136106. doi: 10.1371/journal.pone.0136106 (PMC4542211; doi:10.1371/journal.pone.0136106)

## Slide 1
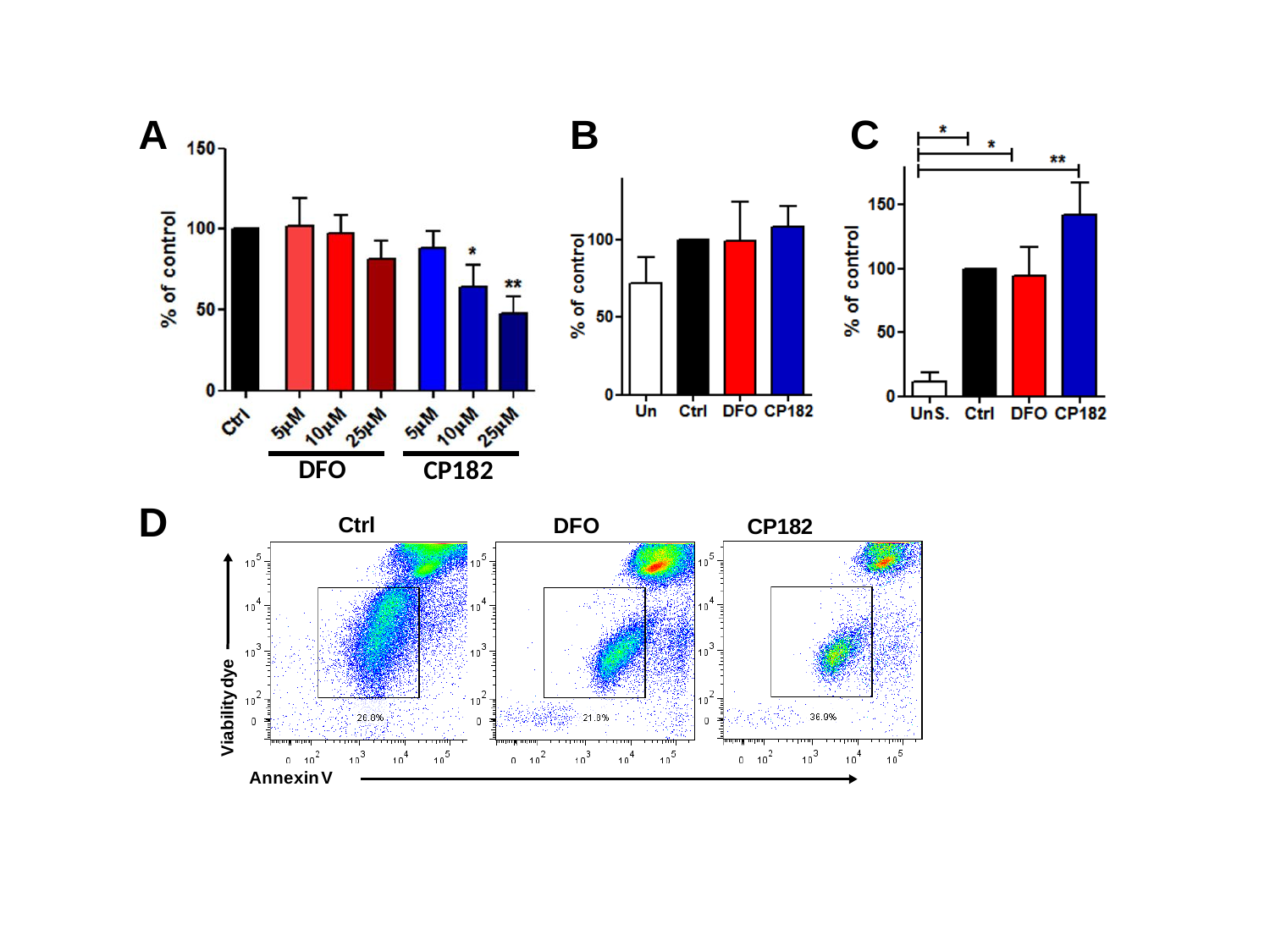

Supplement: S1 Fig — Representative Annexin V stainings in activated CD4+ naïve T cells are exhibited in supplementary figure 1D with no significant differences between groups. (PPTX) [file pone.0136106.s001.pptx]
